# Performance of a Retinal Imaging Camera With On-Device Intelligence for Primary Care: Retrospective Study

**DOI:** 10.2196/70331

**Published:** 2025-07-17

**Authors:** Matthew Silvestrini, Clarissa Lui, Anil Patwardhan, Ying Chen, Tayyeba Ali, Elie Glik, Honglei Wu, Brian Levinstein, Adrianna Wenz, Nathan Shemonski, Lin Yang, Ian Atkinson, Sam Kavusi

**Affiliations:** 1 Verily Life Sciences, 999 Bayhill Dr, San Bruno, CA, 94066, United States, 1 415-786-7939

**Keywords:** diabetic retinopathy screening, retinal camera, retinal imaging, fundus imaging, diabetic retinopathy

## Abstract

**Background:**

Access to screening continues to be a barrier for the early detection of diabetic retinopathy (DR). Primary care–based diabetic retinopathy screening could improve access, but operational challenges, such as cost and workflow management, hamper the widespread adoption of retinal camera systems in primary care clinics in the United States.

**Objective:**

This study aimed to develop and evaluate a retinal screening system suitable for integration into a primary care workflow.

**Methods:**

We developed a nonmydriatic, 45° field imaging retinal camera system, the Verily Numetric Retinal Camera (VNRC; Verily Life Sciences LLC), able to generate high-fidelity retinal images enabled by on-device intelligent features. The VNRC output flows into cloud-based software that accepts and routes digitized images for grading. We evaluated the performance and usability of the VNRC in 2 studies. A retrospective performance study compared the performance of VNRC against a reference camera (Crystalvue NFC-700 [Crystalvue Medical]) as well as the correlation between VNRC capture status and gradability (as determined by ophthalmologist graders). The usability study simulated a primary care setting for a combined cohort of trained and untrained users (corresponding to patients in the simulation) and operators (corresponding to health care personnel in the simulation), where respondents completed a questionnaire about their user experience after attempting to capture images with the VNRC.

**Results:**

In the comparative performance study (N=108, K=206 images), a total of 98.5% (203/206) of images captured by the VNRC were graded as sufficient for clinical interpretation compared to 97.1% (200/206) of Crystalvue NFC-700 images (difference in proportion was 0.015, 95% CI –0.007 to 0.033). In the quality control algorithm evaluation (N=172, K=343 images), we found a positive association (*φ*=0.58, 95% CI 0.45‐0.69) between gradability status (gradable or nongradable) as determined by ophthalmologists and the capture status (recapture not-needed or needed) as determined by the VNRC quality control algorithm. In the usability study (n=15 users and n=30 operators), all participating users (15/15) indicated that they were able to have both eyes screened easily. Most users and operators indicated agreement (from somewhat agree to strongly agree) with statements describing the imaging process as intuitive (15/15, 100% and 29/30, 97%), comfortable (15/15, 100% and 30/30, 100%), and allowing for a positive experience (15/15, 100% and 30/30, 100%), of users and operators, respectively.

**Conclusions:**

Our findings about the performance and usability of this retinal camera system support its deployment as an integrated end-to-end retinal service for primary care. These results warrant additional studies to fully characterize real-world usability across a wider and diverse set of primary care clinics.

## Introduction

Diabetes mellitus (DM) is a growing epidemic that impacts more than 38 million Americans [1], and accounts for a substantial burden to the health care system (approximately US $412 billion) in both direct costs and lost productivity [2]. One of the most common complications associated with DM is diabetic retinopathy (DR), a progressive condition that impacts the microvasculature of the eye and results in irreversible damage to the retina [3,4]. DR, which occurs in approximately 25% of all patients with DM [5], is the leading cause of blindness in the United States and represents a major quality of life burden. Even though studies have found that early detection of DR can mitigate vision loss by over 90% [6], diligent screening of patients with diabetes for DR in the United States remains encumbered by the need for a referral into an ophthalmology clinic. The logistical and financial barriers associated with specialty care are a major cause of noncompliance with the American Diabetes Association screening guidelines [7-9]. These barriers disproportionately impact rural and resource-poor areas, where limitations in the availability of specialists and ophthalmologic equipment may be associated with screening rates lower than 20% [10].

Implementation of DR screening in primary care clinics could improve access [11], and there are retinal camera systems suitable for primary care deployment, such as tabletop Crystalvue (Crystalvue Medical) or Topcon (Topcon Healthcare, Inc) models, and handheld Retinavue (Hillrom), Phelcom (Phelcom Technologies), Remidio (Remidio Innovative Solutions Pvt Ltd), or Volk (Volk Optical) models. Efficient DR screening in primary care, however, faces hurdles. Common issues that impact the consistency of image quality, such as handling small pupil sizes, cataracts, lid and lash occlusions, eye movements, or user positioning, may represent technical challenges for primary care settings. Mydriasis can be another technical challenge; while nonmydriatic imaging is operationally more approachable than mydriatic imaging, it yields some degradation in image quality [12,13]. In addition, the adoption of retinal camera systems in primary care clinics requires resource investments and efficient integration into the clinical workflow. It is therefore unsurprising that DR screening programs tend to succeed in large and well-resourced health organizations that can adopt mydriatic-based systems [14-16], while resource-constrained or small primary-care clinics face difficulties acquiring and operating DR programs [17]. Apart from the direct equipment purchasing costs, training and maintaining dedicated staff (in settings prone to high staff turnover) may become problematic [18]. Furthermore, these environments may often lack optimal infrastructures for electronic health record integration, information technology and Picture Archiving And Communication System systems, and tele-retinal grading services.

These issues are top concerns when tailoring DR screening systems toward primary care. Desirable features in this regard would include relatively affordable cost, as well as the feasibility of integration into existing workflows and of use by nondedicated staff. Yet, it remains imperative to achieve these attributes while maintaining diagnostic accuracy and integrity. DR screening systems need to produce images of sufficient quality to enable ophthalmologists to assess the presence of retinal disease appropriately. Retinal image quality is therefore a necessary condition that correlates with downstream gradability and clinical interpretability.

We report the development and performance characterization of a retinal screening camera system for use in a primary care practice. This system, developed as “Verily Retinal Camera” during the initial investigational period, now termed Verily Numetric Retinal Camera (VNRC; Verily Life Sciences LLC) and provides nonmydriatic, color fundus photos of the eye. Our objectives were threefold: (1) to evaluate the comparative performance of the VNRC against a standard state-of-the-art reference device, the Crystalvue NFC-700 (Crystalvue Medical), for providing high-quality images sufficient for clinical interpretation in a retina image analysis study; (2) to characterize the utility of the VNRC quality control algorithm, investigating the association between the algorithm outputs (scoring the image quality) and human graders’ assessments of whether images were gradable or ungradable; and (3) to assess the usability of the VNRC among potential operators and users in a simulated primary care setting.

## Methods

### Devices

The VNRC and the Crystalvue NFC-700 were used in this study. The VNRC is a nonmydriatic, macula-centered, 45° field imaging retinal camera system that uses a set of proprietary machine learning algorithms to generate high-fidelity composite color posterior chamber images of the eye by capturing and compiling a series of retinal images from each perceived single flash (<200 ms). The camera system is incorporated into a cloud-based software platform that accepts digitized images transferred from the VNRC and has the capacity to store, convert formats, display, and transfer data or imaging data between cameras (Figure S1 in Multimedia Appendix 1).

The Crystalvue NFC-700 camera [19,20] uses a conventional technique [21] to automatically adjust focus and capture the best quality image using a single annulus type of illumination. The image is captured by the built-in color complementary metal-oxide-semiconductor camera module.

### Retrospective Retina Image Analysis Study

This was an analysis of previously collected paired-image acquisitions (discussed further in this study). The objective of this study was to characterize the overall performance of the VNRC, articulated in 2 aims, that is, first, characterize the comparative performance of the VNRC (as an investigational device) against a reference instrument to generate clinically interpretable images, and second, evaluate the utility of the image quality control feature (IQCF) embedded in the camera system (discussed in section “Development and Description of a Retinal Imaging Camera for Primary Care”), by investigating the association between the outputs of this algorithm scoring the quality (ie, the capture status) of the images and image gradeability as determined by ophthalmologists.

#### Study Participants and Image Eligibility

The original trial enrolled 212 participants of at least 22 years of age and who provided informed consent to the protocol; it was conducted in 4 separate phases at 2 sites (Verily Life Sciences South San Francisco headquarters and Diablo Clinical Research). This retrospective study was based on a trial subcohort consisting of non-Verily participants with self-reported history of diabetes (N=172; Figure S2 in Multimedia Appendix 1); this exclusion was to remove sponsor employees as participants in the evaluation of the device.

Analyses were conducted on retinal fundus images collected without mydriasis. For the comparative performance analysis (first aim), eligible images were those from participants who achieved successful image acquisitions without the aid of mydriasis by both cameras (investigational and reference) from both eyes, and for which annotations about clinical interpretability from 3 ophthalmologists were available (n=108 participants). These excluded images captured with only one of the cameras, and also those lacking proper annotations.

For the IQCF utility analysis (second aim), eligible images were those from participants with at least 1 evaluable eye image obtained with the VNRC with a corresponding output from the reference human graders, namely, an annotation as “ungradable” or “gradable” after human inspection (n=172 participants). This excluded those images without proper grader annotation.

#### Study Procedures

Nonmydriatic retinal fundus images were acquired on 2 separate cameras from both right and left eyes of each participant, during a single session. Operators took up to 3 images per eye at their discretion, first on the Crystalvue NFC-700 followed by the VNRC.

Graders received images according to an established protocol. For the Crystalvue NFC-700 camera, graders received the last image acquired for each eye, and for the VNRC, graders received either the first image that passed the quality control feature for each eye, or the last image acquired per eye if no image had passed the quality control feature. This sequencing approach aimed to achieve parity using the highest quality image captured from each camera.

Images from both cameras were presented in random order to 3 board-certified ophthalmologist graders one by one using a grading platform (Figure S3 in Multimedia Appendix 1). Graders were external (non–sponsor-affiliated) professionals who assessed gradability and clinical significance as well as image quality characteristics according to a predefined rubric. The final outcomes were based on the majority vote across 3 graders.

#### Statistical Analyses

##### Aim 1

We compared the performance of VNRC to the reference camera (Crystalvue NFC-700) based on the proportion of acquired images that were determined to be “sufficient quality for clinical interpretation.” This derived binary outcome was based on the majority vote of 3 board-certified ophthalmologists who each reviewed and annotated the same image as “Yes” or “No.” Each participant could contribute up to 1 image from each eye for annotation and subsequent end point evaluation. Duplicate acquisitions of the same eye were not included in any analysis.

The estimated parameter for the end point was the difference in the proportion of images considered sufficient quality for clinical interpretation (ie, Yes), between those from the VNRC camera and those from the Crystalvue NFC-700 camera. Point estimates and 95% CIs for the reported proportions and difference in proportions were based on bootstrap (ie, “cluster” or “block” bootstrap) that accounted for the paired-image design (ie, correlated proportions) and within-participant clustering, as participants provided images from both the right and left eye. This cluster bootstrap was applied as follows:

Define: J=cluster unit = participant, where there may be multiple observation units (ie, eyes) within a participant. The sampling is based on the total number of J clusters.

The first step is to randomly select J number of clusters with replacement. For each cluster selected (with some clusters selected more than once and others not selected at all), all observations (ie, eyes) within that cluster are selected. Original cluster sizes are maintained.

In addition, due to the paired design, whenever an eye image is selected based on the output from 1 camera, the corresponding eye image from the comparative camera is also selected.

A difference in proportion is calculated based on the bootstrapped sample, and the process is repeated B number of times. Our analysis used B=10,000.

The point estimate for the difference in proportion is based on the 50th percentile of the resulting bootstrap distribution. Nonparametric 95% CIs for the difference in proportion were derived based on the 2.5% and 97.5% quantiles of the resulting bootstrap distribution.

##### Aim 2

We investigated the utility of the IQCF based on the association of the scores generated by this algorithm with the classification as “ungradable” or “gradable” by human graders. The algorithm scores image quality, generating an output of “recapture not needed” (eg, sufficient quality) or “recapture needed” (eg, insufficient quality) based on a score threshold. We reported the results from this portion of the study using summaries appropriate for categorical or ordinal data (counts and percentages). The correlation of gradeability status with outputs from the IQCF algorithm was summarized using both a contingency table and the Phi-coefficient.

### Usability Study

#### Study Participants

This study was conducted across two groups (N=45) in a simulated primary care setting: (1) an operator cohort consisting of individuals with health care degrees or licenses (registered nurse, nurse practitioner, Licensed Practical Nurse, and physician assistant; n=15), or some health care training (master’s degree, pharmacy technician, and phlebotomist; n=15), and (2) a user cohort consisting of participants with diabetes without health care training, who were asked to complete the VNRC retinal screening workflow unaided by a technician (n=15). This study was determined to be exempt research that did not require IRB approval.

#### Study Procedures

Operators (group 1) were trained in 2 subgroups. Individuals with health care degrees or licenses and those with some health care training. Training consisted of a visual or auditory slide presentation, demonstration, hands-on activities, and time for the sponsor to answer questions. Approximately half of each subgroup received in-person training, while the other half received one-on-one training from a remote sponsor representative via videoconference. All operators had access to the camera system during training sessions. Training lasted approximately 1 hour. Trained participants experienced a decay period of at least 1 hour and up to 7 hours between their training and test sessions.

The simulated environment (ie, clinic) was equipped with an adjustable height table and all accessories required to use the retinal camera. Participants were asked to perform tasks within representative, naturalistic use scenarios; participants in the operator cohort interacted with pretrained actors (as stand-ins for hypothetical patients) who behaved consistently across simulations to elicit specific responses from operator participants. Moderators used a series of questionnaires around camera ease of use to collect participant feedback.

#### Analysis

Operator and user questionnaires followed Likert scales (from 1=strongly disagree to 7=strongly agree). We analyzed responses using descriptive statistics.

### Development and Description of a Retinal Imaging Camera for Primary Care

**Figure 1.**
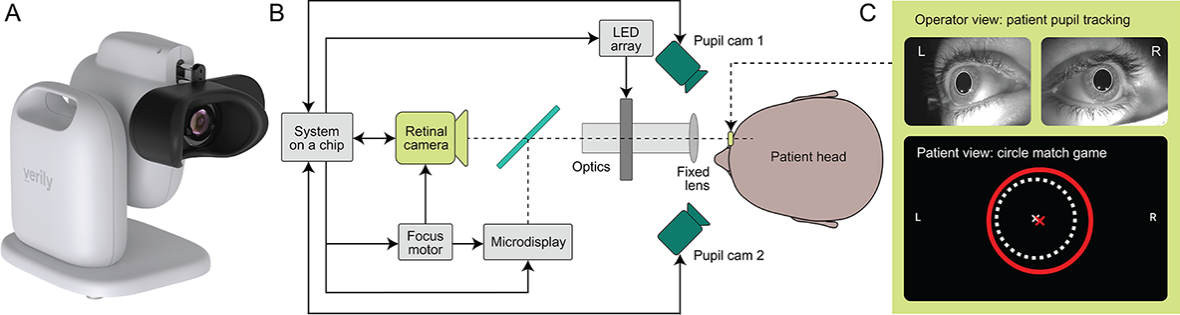
Overview of the Verily Numetric Retinal Camera. (**A**) Illustration of the Verily Numetric Retinal Camera and the (**B**) architecture. (**C**) Operator’s and user’s view during image acquisition. Cam: camera.

The Verily Retinal Service was developed for use in primary care clinics. The VNRC is a lightweight (approximately 6 kg), 45° field imaging camera system consisting of custom electronics, optics, LEDs, and a retinal camera (Figures 1A and 1B). The VNRC has a range of pivot=0° to 45° to adjust for user height, posture, and comfort. The black face rest is light blocking, which enables retinal imaging in bright ambient lighting conditions, and the built-in handle allows for flexibility for camera placement within a clinic space.

The VNRC uses a stereo infrared camera system (pupil camera 1 and 2 in Figure 1B) to achieve proper pupil alignment and facilitate fast image acquisition (120 frames per second [fps]). Users can adjust an image to their best perceived focus with a focus knob, and an interactive game-like interface allows users to optimize proper pupil alignment using small eye or head movements within the face rest, while operators can simultaneously oversee this process to ensure proper eye location (Figure 1C). The focus motor controls both the retinal camera and microdisplay, allowing the user to view an interactive image and video inside the camera. This is similar to an experience in a virtual reality headset, but monocular.

After the initial focus step, users position their head into the VNRC face rest in order to align their eye with the camera lens. Once proper focal length is established by dialing the focus knob, users can confirm by pressing the top of the focus knob. Alternatively, for users unable to use the focus knob, the VNRC can also be brought into focus using an operator-driven setting controlled via the software graphical user interface.

**Figure 2.**
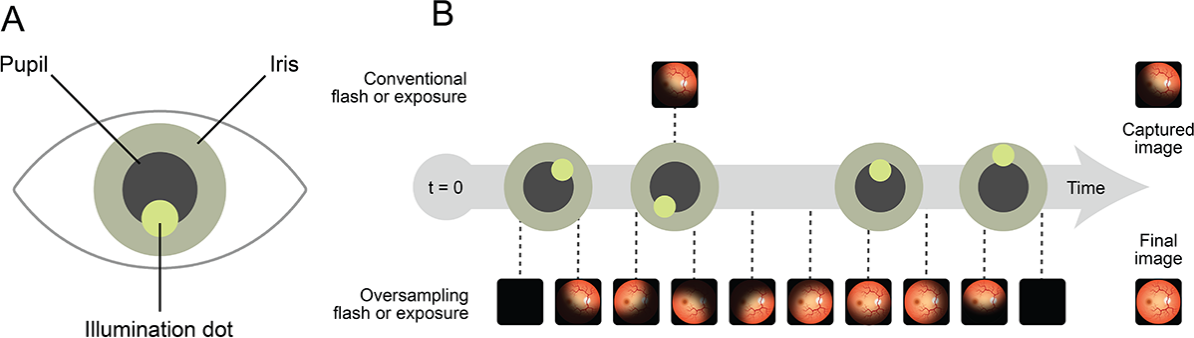
Verily Numetric Retinal Camera’s pupil tracking adaptive illumination. (**A**) Diagram of the eye indicating where the retinal camera projects the illumination dot location onto the pupil area; (**B**) comparison between conventional flash and exposure cameras, where only 1 image is captured and has a higher likelihood of artifacts, and the Verily Numetric Retinal Camera method of oversampling, where a series of images are captured and merged into a final image and hence mitigating the artifacts from any one single image.

The VNRC then uses a proprietary redundant illumination system to automatically collect a series of retinal images (up to 30). This is in contrast to conventional techniques that only capture 1 image frame per flash [21] and is made possible by the use of high-speed high-sensitivity image sensors (120 fps) deeply integrated with the system-on-chip. The flash duration is limited to ensure sufficient quality, minimizing dazzle. Here, the system-on-chip uses an LED array with the camera system to capture a series of full-field (>45°) images of the retina with various illuminations focused on different areas of the crystalline lens of the eye (Figure 2). The proprietary redundant illumination system dynamically adapts to specific eye characteristics, such as pupil size and position, and optimizes a series of retinal illumination configurations, regardless of corneal clarity.

This sequence of flashes, or “burst imaging,” illuminates the entire field of view, occurs within 200 milliseconds, and is perceived as a single flash. The acquisition time falls below the typical latency of the pupil’s response time [22], so that the imaging process is not expected to interfere with natural pupil constriction. The typical burst image set contains many fully illuminated images, with varying levels of artifacts on the images.

**Figure 3.**
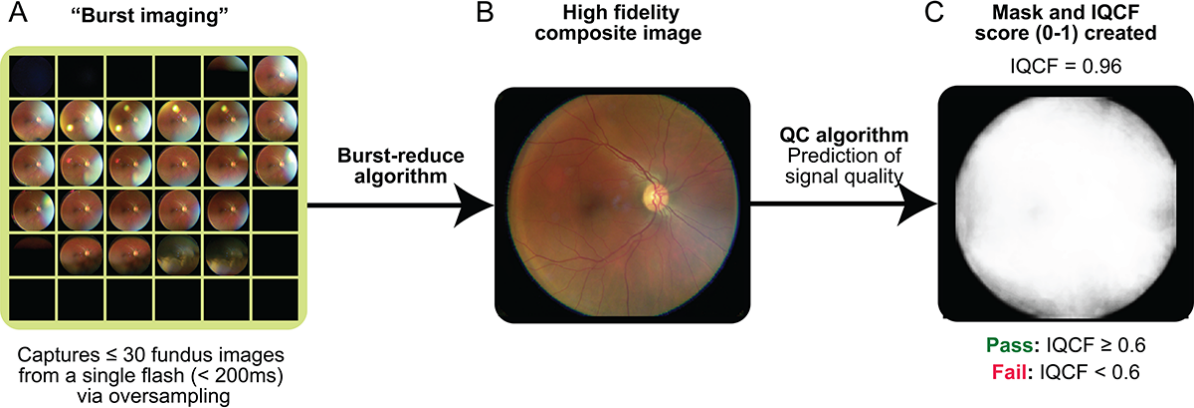
Creation of a single composite image with Verily Numetric Retinal Camera. (**A**) Burst imaging captures up to 30 images from a single flash via oversampling (<200 ms). (**B**) Using a burst reduction algorithm, the Verily Numetric Retinal Camera creates a single high-fidelity composite image. (**C**) A quality control algorithm (the image quality control feature) is then used to predict the signal quality from the retina, and a score is assigned. A passing image quality control feature score indicates that an image is of sufficient quality and does not require recapture. IQCF: image quality control feature; QC: quality control.

The VNRC uses a proprietary “Burst Reduce Algorithm” to generate a single high-fidelity retinal image (Figures 3A and 3B), by merging a variable number of frames. The total number of frames is typically 20 images, but is highly dependent on the presence of artifacts and the amount of eye motion during the camera flash. The algorithm draws similarities from pixel-level high dynamic range imaging [23] but incorporates an assignment of a score layer to separate photons due to retinal reflection from scattered photons due to cataract or cornea reflection, or lid or lash occlusion, after compensating for intra-acquisition gaze shift. It is notable that no fine spatial filtering was applied to enhance the contrast or segment-specific features or pathologies [24,25].

A subsequent algorithm, the IQCF algorithm, then performs a quality control assessment of the resulting composite image to determine if a recapture is needed (Figure 3C). The model was trained to compute areas where the retina is clearly visible, without impaired visibility due to (1) darkness, (2) saturation, (3) blur, or (4) haze. The IQCF algorithm produces a single score per image by calculating the probability that each pixel contributes to that of the retina signal, as represented by an image mask. The algorithm incorporates a fixed cutoff to determine if the image needs to be recaptured. If the score does not exceed this operating point, then the camera will output instructions to the operator that another image acquisition is required. Thus, the VNRC provides real-time image quality characterization information to help reduce the chances that a substandard image is used for clinical interpretation. Importantly, the IQCF does not score image gradeability.

The final output of the VNRC can integrate into a primary care workflow. It feeds into a cloud-based software platform that accepts digitized images transferred from the camera and has the capacity to transfer, store, convert formats, display, and transfer medical device data or medical imaging data between medical devices (Figure S1 in Multimedia Appendix 1). The VNRC also continuously collects metadata such as details on camera use, uptime, and operation in order to allow for real-time error handling. This passively collected metadata is uploaded along with the retinal images.

### Ethical Considerations

This was a retrospective study conducted in a subset of the participants in a prospective technical feasibility trial (Verily protocol 103535; approved by institutional review board [IRB] Western IRB, before initiation, IRB Protocol #20214693) [26]. All participants signed informed consent (Multimedia Appendix 2) approved by the IRB and received nominal compensation for their time ($25 for the screening procedure, an additional $75 for each completed study visit). Personal study-related data were managed in accordance with local data protection law.

## Results

### Comparative Performance of the VNRC Against the Crystalvue NFC-700 Camera

Eye images (K=206) were captured from 108 participants (Table 1) with both the VNRC and the Crystalvue NFC-700 Camera.

**Table 1.** Demographic characteristics of participants in the comparative performance analysis within the retrospective retina image study (as reference, refer to the characteristics of all participants in the original technical feasibility trial in the 2 right columns).

Characteristics	Retrospective study comparative performance (N=108)	Initial technical feasibility trial (N=212)	
		Phase 1‐3 (n=192)	Phase 4 (n=20)
Age (y)			
Mean (SD)	57.8 (13.2)	54.8 (16.6)	49.0 (10.3)
Median (range)	59.5 (23.0‐84.0)	—^a^	—
Sex, n (%)			
Female	60 (55.6)	94 (49)	11 (55)
Male	48 (44.4)	98 (51)	9 (45)
Race, n (%)			
Asian	9 (8.3)	44 (22.9)	<5 (≤5)
Black or African American	18 (16.7)	18 (9.4)	<5 (≤5)
White	76 (70.4)	115 (59.9)	18 (90)
Other	5 (4.6)	15 (7.9)	—
Diagnosed diabetes^b^, n (%)	108 (100)	123 (64.1)	20 (100)
For 0‐15 y	—	35 (18.3)	5 (25)
For 15+ y	—	88 (45.8)	15 (75)

a Not available.

b A post hoc analysis showed 39 (36.1%) participants with mild diabetic retinopathy, 40 (37%) with moderate diabetic retinopathy, and none with severe diabetic retinopathy; 31 (28.7%) had proliferative diabetic retinopathy (based on image evaluation obtained with the reference camera).

**Figure 4.**
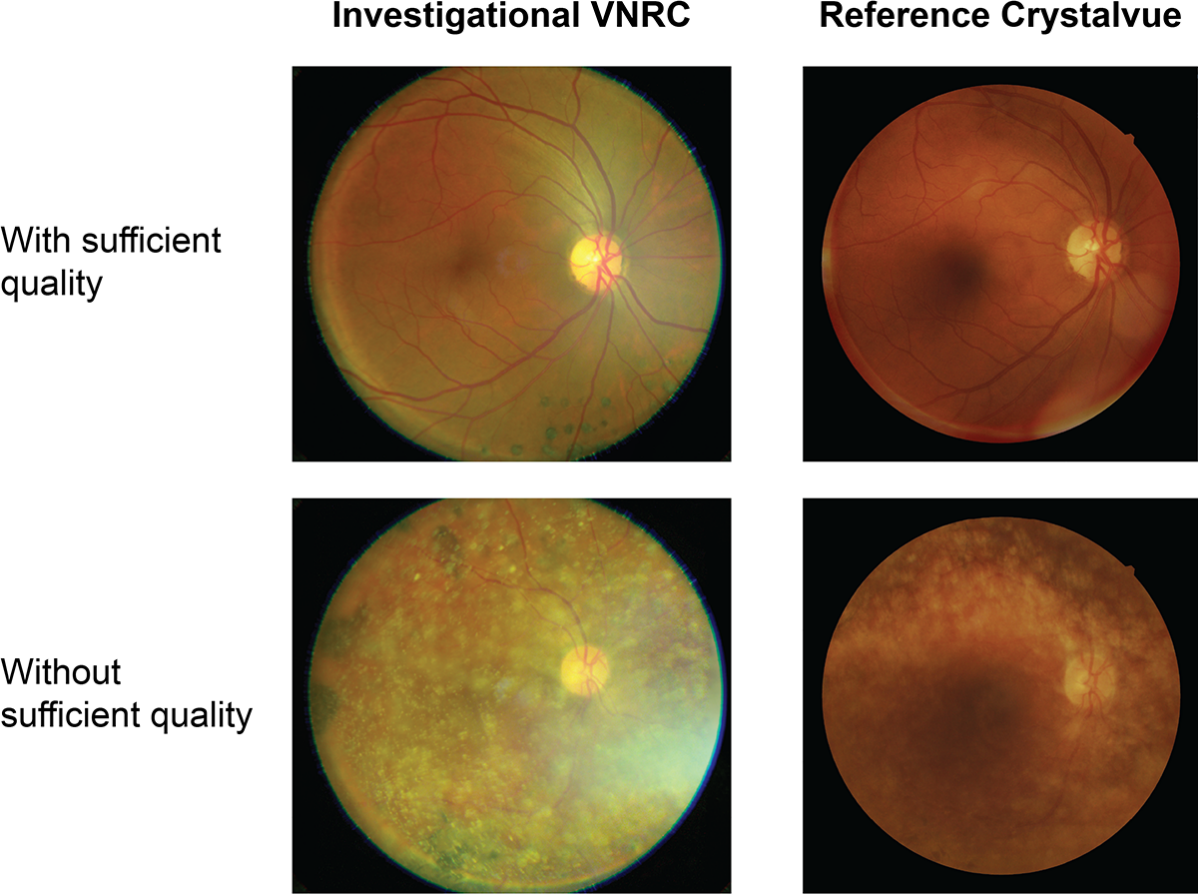
Examples of images with or without sufficient quality for clinical interpretation from each of the cameras in the comparison. VNRC: Verily Numetric Retinal Camera.

The proportion of images of sufficient quality for clinical interpretation was 0.985 (203/206) and 0.971 (200/206) for the VNRC and Crystalvue NFC-700 cameras, respectively. The difference in proportion was 0.015 (95% CI –0.007 to 0.033; Table 2, examples in Figure 4).

**Table 2.** Comparative performance results.

N=108 (K=206 images)	Investigational VNRC^a^ and reference Crystalvue
Images with sufficient quality for clinical interpretation^b^, k (%)	203/206 (98.5) and 200/206 (97.1)
Difference in proportion (95% CI)	0.015 (–0.007 to 0.033)
Uncaptured images, k (n)	3 (2) and 42 (25)

a VNRC: Verily Numetric Retinal Camera.

b Some of the reasons that the images were of insufficient quality: eyelash artifacts, eyelash artifacts, low contrast, or extended areas where detail was lost.

Graders also computed the quality of the images across several specific criteria, for both the VNRC and the reference camera (Tables S1 and S2 in Multimedia Appendix 1), indicating whether the quality of specific image aspects was sufficient for clinical interpretation (note that these were not pooled for a single majority classification; there were 3 separate adjudications for each factor in each image). Graders evaluated the visualization of the optic disc and determined that it was adequate in a majority of images for both devices (in at least 90%, 225/248 of images by the VNRC and in at least 93%, 195/209 of the reference). There were similar results in the classification of macula visualization (deemed appropriate in at least 92%, 229/248 of VNRC images and 83%, 175/209 of reference images), and the visualization of the retinal vessels (appropriate in at least 94%, 233/248 of the VNRC images and 92%, 194/209 of reference images). Graders also found a majority of images to be of sufficient quality regarding key imaging features, such as adequate focus (in at least 91%, 228/248 of VNRC images and 88%, 185/209 of reference images), appropriate brightness (in at least 94%, 213/248 and 198/209 of images, for both), adequate field of view (in at least 91%, 228/248 and 191/209 of images, for both), no significant image defects (in at least 91%, 227/248 and 92%, 194/209), no small pupil interference (at least 89%, 222/248 and 87%, 183/209), and no ocular media opacity (in at least 73%, 183/248 and 85%, 178/209 of VNRC and reference images, respectively).

### Performance of the IQCF

We found a moderate association (*φ*=0.58) between ophthalmologists’ assessments of a retinal image’s gradeability and the IQCF algorithm’s scoring of capture status (Table 3).

**Table 3.** Contingency table of image quality control factor scores of image quality and gradeability assessments determined by ophthalmology graders.

IQCF^a^ score (N=172; K=343 images)	Graders’ rating, n (%)		Total
	Nongradable	Gradable	
Recapture needed (IQCF=not pass)	20 (40^b^)	30 (60)	50
Recapture not needed (IQCF=pass)	1 (0.3)	292 (99.7)	293

a IQCF: image quality control factor.

b Percentages reflect the total number of image quality control factor classifications that were determined to be nongradable or gradable during human assessment (ie, graders’).

**Figure 5.**
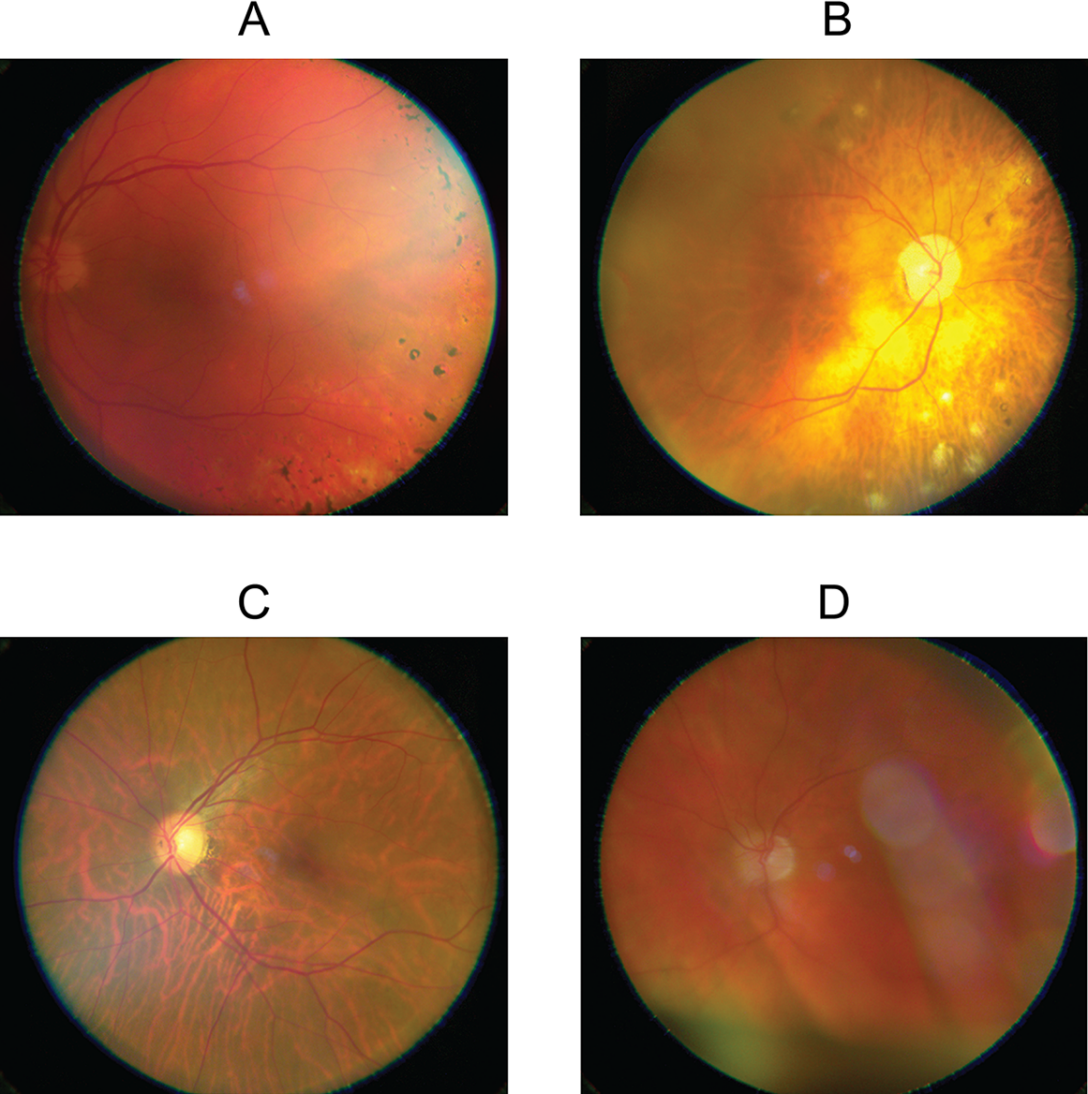
Example images of different image quality control feature scores and human assessments. (**A**) Image quality control feature “pass” with acceptable image quality, but ungradable due to optic disc shift. (**B**) Image quality control feature “not pass” with poor image quality, but gradable due to visible lesions. (**C**) Image quality control feature “pass” and gradable. (**D**) Image quality control feature “not pass” and ungradable, due to eyelash artifacts.

Overall, the IQCF scored 50 images as needing recapture, but the human assessment was “gradable” for 60% (30/50) of these. Conversely, the vast majority of the images scored by the IQCF as not needing recapture, 292 out of 293 (99.7%), were found “gradable” by human assessment. Refer to Figure 5 for examples of images with concordant and discordant IQCF scoring and human assessment.

### User Research Study

There was agreement among 100% (15/15) of the participating users in the simulated clinic environment that they were able to have both eyes screened easily. In addition, they somewhat agreed or strongly agreed (rating of 5 to 7 on a 1‐7 Likert scale) with statements indicating that they were confident in knowing how to complete the screening after watching the video, that they found it intuitive to set themselves up with the camera properly, felt comfortable while completing the screening, and had a positive experience with the camera (Table 4, Table S3 in Multimedia Appendix 1).

**Table 4.** Summary questionnaire results for simulated users (no health care training).

Scale and question	Users (n=15), n (%)
Likert scales (range 1-7)^a^, responses>4	
Q1: I felt confident that I knew how to do the screening after watching the video	15 (100)
Q2: I found it intuitive to get myself set-up with the camera properly	15 (100)
Q3: I felt comfortable when doing the screening	15 (100)
Q4: I had a positive experience using the camera	15 (100)
Binary (yes or no), response “yes”	
Q5: I had both my eyes screened easily	15 (100)

a Likert scale, where 1=strongly disagree and 7=strongly agree.

All participating operators under simulated conditions (30/30, 100%), who had either health care degrees, licenses, or some training somewhat agreed or strongly agreed (ratings of 5 to 7 on a 1‐7 Likert scale) with statements indicating that they felt comfortable completing the screening, had a positive experience while using the camera, found training easy and useful to understand, found it easy to capture a retinal image with the camera, and found the camera easy to clean (Table 5, and Table S4 and S5 in Multimedia Appendix 1). A majority (23/30) felt that hands-on help for users was probably not needed, and approximately half (14/30) felt that they would not have to apply their relevant clinical training to complete specific tasks.

**Table 5.** Summary questionnaire results for simulated operators (with health care licenses or training).

Questions on Likert scale (range 1-7)^a^	Participants with responses >4, n (%)		
	Health care degree or license (n=15)	Health care training (n=15)	Pooled (n=30)
Q1: I felt comfortable when doing the screening	15 (100)	15 (100)	30 (100)
Q2: I feel like I needed to provide hands on help for the patient	1 (7)	6 (40)	7 (23)
Q3: I had a positive experience using the camera	15 (100)	15 (100)	30 (100)
Q4: I found the training easy to understand and useful	15 (100)	15 (100)	30 (100)
Q5: I found it easy to capture a retinal image with the camera	15 (100)	15 (100)	30 (100)
Q6: I had to apply my relevant clinical training to complete specific tasks	8 (53)	8 (53)	16 (53)
Q7: I found the camera user interface intuitive and easy to understand	15 (100)	14 (93)	29 (97)
Q8: I found the camera easy to clean	15 (100)	15 (100)	30 (100)

a Likert scale, where 1=strongly disagree and 7=strongly agree.

Most of the issues reported by users related to involuntary clicks back and forth in the user interface, particularly during the “focus image” steps. These issues, at most, caused delays (not failures) in the image capture process.

## Discussion

### Principal Findings

We report here the results of a series of analyses to characterize the performance and usability of a new retinal camera system aimed for implementation in primary care settings. The performance was satisfactory in 2 main aspects. First, the VNRC performed at comparable levels to a reference camera for the generation of quality retinal images; the numeric difference in the percentage proportion of images with quality to be clinically interpretable was low (0.015), and the CI for that difference straddled 0, indicating a likelihood that there was no difference between the 2 devices. Further reinforcing the main results, gradability across a variety of image quality metrics appeared numerically similar across VNRC and the reference camera. Second, the quality-scoring outputs of the quality control algorithm embedded in the system showed a moderate association (*φ*=0.58) with the classification of images as “gradable” or “ungradable” by human graders. Furthermore, operators and users (ie, individuals with and without previous health care training) found the system to be generally intuitive and approachable to use, allowing them to feel comfortable performing image captures on their own (at least 95%, 44/45 of survey participants agreed to the corresponding statements).

Our findings indicate that VNRC can perform at the level of a standard tabletop retinal camera system. Thus, the VNRC may provide a balance of features that could mitigate some of the reservations from primary care providers to the adoption of DR screening programs. Across both primary care and specialty clinics, tabletop equipment may be the highest quality option for retinal imaging [27]; however, high direct costs and the resource and space demands of optimal installation (ie, dedicated darkened room) act as deterrents, particularly in underresourced environments. While handheld and smartphone-based cameras overcome these aspects, reports are mixed regarding the quality of their image capture and their ease of successful use for DR screening (for instance, the skill and finesse required to obtain proper eye alignment [13,28], particularly when imaging is performed without pharmacologic pupil dilation [13,18,29-31]. Most of the images excluded from the comparative analysis (for lack of bilateral counterparts) were in the reference camera group (probably due to the inability of that camera to capture images for pupil sizes below 3.5 mm). The rates of ungradable images we observed are encouragingly low, considering that other studies with mydriatic devices (where they would be expected to be lower) have reported approximately 6% [32], or ranges from 0% to approximately 28% with mydriatic cameras (when grading was done via algorithm) [33]. A possible downside of producing a high rate of ungradable images (which can be due to a variety of causes, such as small pupil size, eye pathologies) may be an inflation of referrals that can overwhelm downstream eye care services and reduce the overall cost-effectiveness of teleophthalmology programs [18,30]. The VNRC has advantages closely associated with handheld systems in cost, maneuverability, relatively low weight (10‐20 kg lighter than other tabletop systems), and relatively low space demands. Our results suggest that this system can produce images of quality comparable with expensive and more sophisticated tabletop equipment, overall and across specific image metrics, possibly reaching a balance between operational requirements and quality performance, which is particularly well-suited to primary care clinics.

Our results also indicate that the quality control feature within the VNRC system, the IQCF, functions as intended and could effectively filter out the intake of undesirable, poor-quality images into practical clinical workflows. Capturing retinal images of quality is a necessary requisite for the subsequent clinical utility of those images during diagnosis; entering a high proportion of low-quality images could render a clinical workflow inefficient and impair effective care. The approach presented in this work was to embed an algorithm to score image quality in real time, prompting users to discard low-quality images (those below a score threshold) and recapture before entering an image into the clinical workflow. Thus, it was important to establish the correlation between the proportion of retinal images passing the VNRC’s IQCF threshold and downstream assessments of gradability according to ophthalmic graders. We found a moderate correlation, as nearly all the images cleared by the IQCF as “not needing recapture” were indeed found “gradable” by human assessment. The IQCF scored noticeably more images as “needing recapture” than human graders deemed “ungradable” (ie, 60%, 30/50 of images that IQCF labeled as needing recapture were actually deemed gradable during human assessment). This is probably due to the fact that the IQCF is a visibility measure, with an output that is pathology-independent; in contrast, human graders may grade images if a lesion is clearly identifiable, even in a context of low overall quality or visibility that the IQCF probably would “not pass.” This excess of images with “recapture needed” scores, however, may not represent a major practical problem, since image recapture in real time may not be a burdensome procedure. Ultimately, this ensures that the IQCF facilitates an inflow of quality images, without erring in a direction that would create backflows or inefficiencies, impairing practical clinic workflows after the fact.

Our third major result showed that participants found that handling the VNRC system was easy and intuitive and felt comfortable with it. This addresses another relevant concern for primary care practice managers considering retinal screening systems, namely, the actual or perceived need for dedicated trained staff. Furthermore, systems with which users and operators experience repeated lack of success in producing images of sufficient quality may undermine confidence. In turn, this can nudge personnel toward lower usage [18] and depress the cost-effectiveness of a screening program. Our survey results indicate that the VNRC could be an approachable system that mitigates usability barriers.

While our analyses yielded promising results, the characterization of this new DR screening system had some limitations, largely related to the generalizability of our findings. First, the images for the comparative performance analysis were collected first with the VNRC, followed by the reference camera, in order to maintain internal consistency with the larger study from which this retrospective analysis was undertaken [26]. Therefore, we cannot discount the possibility of experimental bias related to the order of measurement (it could be a learning effect or a fatigue effect) that could have influenced the results. Second, our analyzable dataset for the comparative analysis images excluded participants without complete bilateral, nonmydriatic image sets and annotations, which could have exerted some selection bias toward better-quality cases; while that effect would be expected to some extent for both groups in the comparison, it may have had a differential effect that we cannot discount. Third, we cannot interpret the potential impact of not-captured images in our comparative performance analysis. Some of those noncaptures may correspond to smaller pupil sizes, since the instructions for use for Crystalvue NFC-700 require a minimum pupil size of 4 mm. Therefore, different study conditions (eg, dimming ambient light in order to dilate pupil for an attempted recapture) may yield different comparative results from the ones in this report. Fourth, the VNRC IQCF would encourage image recaptures for low-quality scores in an actual clinic setting, until the operator has a total of 3 eye images or one of the images scores above the threshold. In this study, graders received image captures according to a prespecified sequence that may not have consistently maximized quality. Therefore, future studies are warranted to investigate performance across a more diverse set of conditions. For instance, future postmarket analyses could evaluate performance relying on the images with the top IQCF score across all eye captures, or in different environmental setups (such as lighting), to better reflect performance in real-clinic conditions. Another area for future study is the characterization of variability in larger cohorts, more widely representative of primary-care screening populations. Importantly, disease characteristics (burden and type of disease) may impact the relative performance in terms of gradeability and the association of image quality with gradeability. Finally, we did not collect information on visual acuity from the participants in the user study; while results were overall favorable, visual acuity (or lack thereof) may affect user-device interactions; therefore, it will be worthwhile to gather appropriate information in future studies to better understand it.

### Conclusions

In summary, barriers to primary case-based DR screening overall are multifactorial [18,34,35]. Providers tend to perceive that rigorous and cost-effective implementation of teleophthalmology in primary care settings is expensive and difficult, demanding restructuring of work processes and increasing the burden on clinic staff [18,36]. Our results indicate that the VNRC system could mitigate some of these issues, particularly in underresourced environments. It has the positive operational characteristics akin to handheld systems, produces images of quality comparable with standard tabletop retinal cameras, and is able to optimize the inflow of quality images into clinical workflows. In addition, users note that they are able to handle the system and produce usable images with ease. Furthermore, the transfer and flow of the digital output are adaptable to typical primary care workflows. These results support considering this system as an integrated end-to-end retinal service suitable for primary care and warrant additional studies across a wider and diverse set of primary care clinics. Novel DR screening systems that address primary care adoption barriers may represent an advance toward more widespread access, with the potential to curtail rates of severe disease progression at the population level and ultimately contribute to better patient outcomes.

## Supplementary material

10.2196/70331 Multimedia Appendix 1 Additional methods and results.

10.2196/70331 Multimedia Appendix 2 Consent form.

## Abbreviations

DM: diabetes mellitus
DR: diabetic retinopathy
fps: frames per second
IQCF: image quality control feature
IRB: institutional review board
VNRC: Verily Numetric Retinal Camera
