# Supplementary material for: Performance of a Retinal Imaging Camera With On-Device Intelligence for Primary Care: Retrospective Study
Source: JMIR Form Res. 2025 Jul 17;9:e70331. doi: 10.2196/70331 (PMC12289297; doi:10.2196/70331)
Supplement: Multimedia Appendix 1 [file formative-v9-e70331-s001.docx]

##

**MULTIMEDIA APPENDIX 1: METHODS and RESULTS**

**Figure S1. EHR integration of the VNRC system.**



**Figure S2. Participant flow into the performance and usability studies.**

1. **Performance Studies**

**

**

1. **User Research**

**

**

**Figure S3. Comparative Performance Study Methods**

**.**

**Table S1. Comparative Performance Image Quality.**

| **Reference (K=209)** | **Optic Disc** | **Macula** | **Retinal Vessels** |
| --- | --- | --- | --- |
| Number of Images sufficient for clinical interpretation (Range across all graders) | 195-206 | 175-199 | 194-206 |
| % Images sufficient for clinical interpretation (Range across all graders) | 93.3-98.6% | 83.7-95.2% | 92.8-98.6% |
|  | | | |
| **VNRC (K=248)** | **Optic Disc** | **Macula** | **Retinal Vessels** |
| Number of Images sufficient for clinical interpretation (Range across all graders) | 225-234 | 229-238 | 233-237 |
| % Images sufficient for clinical interpretation (Range across all graders) | 90.7-94.4% | 92.3-96.0% | 94.0-95.6% |

**Table S2. Comparative Performance Descriptive Statistics.**

| **Reference (K=209)** | **Adequate Focus** | **Appropriate Brightness** | **Good Field View Identification** | **No Image Defects** | **No small pupil Interference** | **No Ocular Media Opacity** |
| --- | --- | --- | --- | --- | --- | --- |
| Number of Images sufficient for clinical interpretation (Range across all graders) | 185-191 | 198-202 | 191-207 | 194-208 | 183-206 | 178-207 |
| % Images sufficient for clinical interpretation (Range across all graders) | 88.5-91.4% | 94.7-96.7% | 91.4-99.0% | 92.8-99.5% | 87.6-98.6% | 85.2-98.1% |
|  |  |  |  |  |  |  |
| **VNRC (K=248)** | **Adequate Focus** | **Appropriate Brightness** | **Good Field View Identification** | **No Image Defects** | **No small pupil Interference** | **No Ocular Media Opacity** |
| Number of Images sufficient for clinical interpretation (Range across all graders) | 228-233 | 213-237 | 228-240 | 227-239 | 222-240 | 183-240 |
| % Images sufficient for clinical interpretation (Range across all graders) | 91.9-94.0% | 94.7-96.7% | 91.9-96.8% | 91.5-96.4% | 89.5-96.8% | 73.8-96.8% |

**Table S3. Summary of questionnaire responses on usability from the cohort of simulated users (n=15).**

| **Simulated User Population:**  Participants with Type II Diabetes | **Usability Questionnaire**  (Q1-4: 1-7 on Likert scale, where 1=’strongly disagree’ and 7=’strongly agree’, Q5: 1= yes and 0= no) | | | | |
| --- | --- | --- | --- | --- | --- |
|  | Q1: I felt confident that I knew how to do the screening after watching the video | Q2: I found it intuitive to get myself set-up with the camera properly | Q3: I felt comfortable when doing the screening | Q4: I had a positive experience using the camera | Q5: I had both my eyes screened easily |
| 1 | 6 | 7 | 5 | 6 | 1 |
| 2 | 7 | 7 | 7 | 7 | 1 |
| 3 | 7 | 7 | 6 | 7 | 1 |
| 4 | 6 | 6 | 7 | 7 | 1 |
| 5 | 7 | 7 | 7 | 7 | 1 |
| 6 | 6 | 7 | 7 | 7 | 1 |
| 7 | 6 | 6 | 7 | 7 | 1 |
| 8 | 7 | 7 | 7 | 7 | 1 |
| 9 | 7 | 7 | 7 | 5 | 1 |
| 10 | 6 | 6 | 7 | 6 | 1 |
| 11 | 7 | 7 | 7 | 7 | 1 |
| 12 | 7 | 6 | 7 | 7 | 1 |
| 13 | 7 | 7 | 7 | 7 | 1 |
| 14 | 7 | 7 | 7 | 7 | 1 |
| 15 | 7 | 7 | 7 | 7 | 1 |
| **Median** | **7** | **7** | **7** | **7** | **1** |
| **Range** | **6 - 7** | **6-7** | **5-7** | **5-7** | **1** |

**Table S4. Summary of questionnaire responses on usability from the cohort of simulated operators with healthcare degrees or licenses (n=15).**

| **Simulated Operator Population:**  Participants with healthcare degrees, licenses, or some healthcare training | **Usability Questionnaire**  (Q1-8: 1-7 on Likert scale, where 1=’strongly disagree’ and 7=’ strongly agree’ | | | | | | | |
| --- | --- | --- | --- | --- | --- | --- | --- | --- |
|  | Q1: I felt comfortable when doing the screening | Q2: I feel like i needed to provide hands on help for the patient | Q3: I had a positive experience using the camera | Q4: I found the training easy to understand and useful | Q5: I found it easy to capture a retinal image with the camera | Q6: I had to apply my relevant clinical training to complete specific tasks | Q7: I found the camera user interface intuitive and easy to understand | Q8: I found the camera easy to clean |
| 1 | 5 | 1 | 6 | 5 | 7 | 4 | 6 | 7 |
| 2 | 7 | 1 | 7 | 7 | 5 | 1 | 6 | 7 |
| 3 | 6 | 1 | 7 | 7 | 7 | 3 | 5 | 7 |
| 4 | 7 | 2 | 7 | 7 | 7 | 7 | 7 | 7 |
| 5 | 7 | 5 | 6 | 7 | 5 | 5 | 6 | 7 |
| 6 | 7 | 3 | 7 | 7 | 7 | 4 | 7 | 7 |
| 7 | 7 | 2 | 7 | 7 | 7 | 4 | 7 | 7 |
| 8 | 7 | 1 | 7 | 7 | 7 | 6 | 7 | 7 |
| 9 | 7 | 1 | 7 | 7 | 7 | 6 | 7 | 7 |
| 10 | 6 | 4 | 7 | 7 | 6 | 7 | 7 | 7 |
| 11 | 7 | 1 | 7 | 7 | 7 | 7 | 7 | 7 |
| 12 | 6 | 3 | 5 | 7 | 6 | 1 | 6 | 7 |
| 13 | 7 | 1 | 7 | 5 | 7 | 7 | 7 | 7 |
| 14 | 7 | 1 | 7 | 7 | 7 | 1 | 7 | 7 |
| 15 | 6 | 6 | 7 | 7 | 6 | 6 | 7 | 7 |
| **Median** | **7** | **1** | **7** | **7** | **7** | **5** | **7** | **7** |
| **Range** | **5-7** | **1-6** | **5-7** | **5-7** | **5-7** | **1-7** | **5-7** | **7** |

**Table S5. Summary of questionnaire responses on usability from the cohort of simulated operators with some healthcare training (n=15).**

| **Simulated Operator Population:**  Participants with some healthcare training | **Usability Questionnaire**  (Q1-8: 1-7 on Likert scale, where 1= ‘strongly disagree’ and 7= ‘strongly agree’ | | | | | | | |
| --- | --- | --- | --- | --- | --- | --- | --- | --- |
|  | Q1: I felt comfortable when doing the screening | Q2: I feel like I needed to provide hands on help for the patient | Q3: I had a positive experience using the camera | Q4: I found the training easy to understand and useful | Q5: I found it easy to capture a retinal image with the camera | Q6: I had to apply my relevant clinical training to complete specific tasks | Q7: I found the camera user interface intuitive and easy to understand | Q8: I found the camera easy to clean |
| 1 | 7 | 2 | 7 | 7 | 7 | 1 | 7 | 7 |
| 2 | 6 | 5 | 7 | 7 | 6 | 6 | 5 | 7 |
| 3 | 6 | 3 | 7 | 7 | 7 | 7 | 6 | 7 |
| 4 | 7 | 5 | 7 | 7 | 7 | 5 | 6 | 7 |
| 5 | 7 | 2 | 7 | 7 | 7 | 4 | 7 | 7 |
| 6 | 5 | 5 | 6 | 6 | 6 | 5 | 5 | 6 |
| 7 | 7 | 1 | 7 | 7 | 7 | 7 | 7 | 7 |
| 8 | 7 | 1 | 7 | 7 | 7 | 1 | 7 | 7 |
| 9 | 7 | 1 | 7 | 7 | 7 | 3 | 7 | 7 |
| 10 | 7 | 2 | 7 | 7 | 7 | 1 | 7 | 7 |
| 11 | 7 | 6 | 7 | 7 | 7 | 5 | 6 | 7 |
| 12 | 7 | 5 | 7 | 7 | 5 | 6 | 7 | 7 |
| 13 | 5 | 2 | 5 | 5 | 5 | 3 | 4 | 7 |
| 14 | 7 | 6 | 7 | 7 | 7 | 7 | 7 | 7 |
| 15 | 7 | 1 | 7 | 7 | 6 | 2 | 7 | 6 |
| **Median** | **7** | **2** | **7** | **7** | **7** | **5** | **7** | **7** |
| **Range** | **5-7** | **1-6** | **5-7** | **5-7** | **5-7** | **1-7** | **4-7** | **6-7** |
