# Supplementary material for: Performance of a Retinal Imaging Camera With On-Device Intelligence for Primary Care: Retrospective Study
Source: JMIR Form Res. 2025 Jul 17;9:e70331. doi: 10.2196/70331 (PMC12289297; doi:10.2196/70331)
Supplement: Multimedia Appendix 2 [file formative-v9-e70331-s002.pdf]

RESEARCH PARTICIPANT CONSENT FORM

STUDY TITLE: Verily Retinal Camera Feasibility Study

STUDY PROTOCOL NO.: 103535
IRB Protocol #20214693

SPONSOR: *Verily Life Sciences LLC*

[redacted]

[redacted]

[redacted]

[redacted]

[redacted]

SUMMARY

You are being invited to participate in a research study. The purpose of this consent form is to help you decide if you want to participate in this research study. It explains what will happen during the study, why it is being done, and your rights as a research participant.

You have the right to know what will happen during this study, as well as the possible risks and benefits of participating in this study. Signing the last page of this document will mean that you agree to participate. During the study, you will be told of any important new findings about the study device. You can use this information to decide about continuing in the study. You can stop participating in the study at any time.

This consent form may contain scientific concepts or other words that are unfamiliar. Please ask the study staff to explain anything that you do not clearly understand. You should not join this research study until all of your questions are answered and you are comfortable with participating in this research study.

To summarize, if you sign this form, you are giving your consent to participate in this study. Up to 1000 research participants will be in this study at up to 4 sites in the U.S.

PURPOSE OF THE STUDY
By signing this form, you agree to take part in a study where you will have images of the back of your eye (retina) taken by an investigational retinal camera (Materials). Verily may use the data collected in this study to evaluate and improve the materials in this study including, but not limited to the study device, instructional materials, and related technologies.

The purpose of this study is to capture retinal images and collect feedback for the purposes of device development.

This information may be used for future research and the development of future devices.

The device used in this study is investigational, which means it has not been cleared or approved by the US Food and Drug Administration ("FDA").

By signing this form, you agree to follow all instructions provided to you by the study staff. You will not take apart, remove parts, disassemble, attempt to replicate or copy the device or its associated materials, or allow anyone else to do so. You will not sell, loan, or give away the device or its associated materials to anyone else. If any of the provided materials are lost or stolen, you must contact the study staff immediately. Verily retains title, ownership, and all rights to the materials and anything else that you are provided in connection with the study.

When you come to Verily's onsite location as part of the study, you must comply with any instructions provided by Verily during your visit.

PROCEDURES
If you choose to take part in this study, the following procedures may take place.

Screening and Informed Consent
- Verify your identity
- Informed Consent discussion and sign written or electronic Informed Consent Form
- Confirm you meet all eligibility requirements
- Demographic Information
- Medical history and medications

If you participate in multiple study sessions, we may need to repeat Screening Activities.

If you do ***not*** meet all the inclusion criteria or meet any exclusion criteria, you will be exited from the study.

<u>Inclusion Criteria</u>

- At least 22 years of age
- Able and willing to sign the written Informed Consent Form
- Able and willing to use the study device during the study visit
- US resident

<u>Exclusion Criteria</u>

- Participant is contraindicated for imaging by fundus imaging system used in the study; e.g., Participant is hypersensitive to light (side effect of medication or due to unknown etiology)
- Self-reported history of epilepsy or light-induced seizure
- Self-reported allergy to rubber and/or foam
- Any other condition or situation that, in the opinion of the Investigator, makes the subject inappropriate for participation in the study

<u>Study Activities:</u>
Upon successful completion of the Screening Activities, you will continue to the Study Visit. This study visit may be on the same day as the Screening Activities.

The Verily Retinal Camera (VRC) is an investigational device, so no diagnostic value can be discerned from measurements taken during the Study Visit.

If you choose to participate in multiple study activity sessions subsequent visits may be scheduled for later dates.

During the Study Visit, the following may occur:

1. The Investigator (or designee) may ask for the following information or do the following procedures
    a. Document the color of your eyes
    b. Measure your pupils
    c. The study staff may use a standard ophthalmic machine to estimate your refractive measurement (for example, nearsightedness and astigmatism)
    d. The study staff may perform additional assessments of your eyes, such as a slit lamp examination and visual acuity measurement
    e. The study staff may use a ruler, or other measurement tool, to take anthropometric measurements, such as the distance between your pupils
    f. You may have up to 5 images taken per eye with each investigational VRC
    g. You may have up to 5 images taken per eye with up to 3 standard

ophthalmic fundus cameras

h. You may be asked to complete a survey regarding your experience with the device(s)
i. Additional study staff may be present to provide technical support of the study devices
j. With your written consent, photographs or video may be taken during use of the retinal camera. Best efforts will be made to de-identify you, however it may be possible to identify you in the photographs. Photographs and video are optional, and you may continue to participate in the study if you decline to have photographs and/or video taken.
k. You may be asked to complete surveys about your experience.

STUDY DURATION

Your participation in this study will be approximately 1 to 1.5 hours for each study visit. You may be eligible, but are not required, to participate in multiple study visits.

RISKS AND DISCOMFORTS

This study is considered a non-significant risk device study. The types of risk you may experience are similar to those of cameras that take pictures of the back of the eyes.

- Temporary light sensitivity
- Temporary (less than 5 minutes) discoloration of vision from camera flashes
- Temporary (less than 5 minutes) eye strain due to viewing of display images
- Temporary headache
- Temporary blurred vision
- Temporary eye irritation

Lastly, potential risks due to data breach can cause the de-identified test data to be lost, compromised, or accessed by unauthorized parties.

There may be other risks or side effects that are not known at this time.

PREGNANCY AND BREASTFEEDING RISKS

There may be unknown risks to pregnant women, an embryo or fetus, or to nursing infants.

If you become pregnant during the study, you must tell the study staff immediately. He/she will advise you on further medical attention should this be necessary.

NEW INFORMATION

You will be told about any new information that becomes known to researchers that might change your decision to be in this study. You may be asked to sign a new consent

form if this occurs.

BENEFITS
There is no direct benefit to you for participating in this study; however, the information obtained in this study may be helpful to others in the future.

You will not have any rights to licensing or receive financial compensation from any products that may result from participating in this study.

COSTS
The study procedures will be provided to you at no cost. You will not incur any additional costs by agreeing to participate in this study.

PAYMENT FOR PARTICIPATION

You will be paid $25 for completing screening activities.

If you are selected to participate, you will be paid $75 for each completed study visit.

Payments will be processed using a third party (which means they are independent from the study Sponsor) payment vendor called Hyperwallet. Hyperwallet will know of your participation in this study. You will set up an account directly with Hyperwallet and will be given the option to receive payment through direct bank transfer or through a physical payment card that will be mailed to you. This payment card may be used anywhere Visa is accepted. Your payment card will be remotely loaded with cash based on the study procedures you've completed.

Payment cards may be subject to standard fees and other terms as described in Hyperwallet's cardholder agreement. It may take up to 5 weeks to receive payment.

You will need to provide Hyperwallet with your name, email address, mailing address, and your date of birth. If you choose electronic bank transfer, you will need to provide your banking information. Your information will be kept secure and confidential, as described in the "Confidentiality" section.

Once you complete your first compensated activity, you will receive an "account activation email" from Hyperwallet. You'll need to activate your account and select your payment preferences within 180 days from the first payment issue date. After 180 days the payment will expire and will be considered forfeit -- if this happens the payment will not be reissued.

Upon your completion of the study, compensation support from Verily will terminate after [60-90] days from your study exit. During this time you should raise any compensation questions that you have and we will quickly work to resolve all open issues. If you elected to be paid with a payment card, we strongly recommend spending

your balance within this window. Further compensation support will not be provided outside of this period.

You are being compensated for your participation in this study. If the total payment for your participation in Verily Life Sciences LLC studies is greater than $600 in a calendar year, it will be reported to the Internal Revenue Service (IRS) as income and your social security number may need to be collected. In this case, you must provide your tax reporting documentation to receive payment.

ALTERNATIVE TREATMENT

Since this study is not therapeutic in nature, the only alternative to participation is not to participate in this study.

CONFIDENTIALITY

Data that could be used to identify you

Information about you or provided by you during the research will not be disclosed to others without your written permission, except if it is necessary to protect your rights or welfare (for example, if you are injured and need emergency care); or if it is required by law. The information about you will be kept confidential throughout the study. A unique participant identification code will be used when collecting study data.

Authorized representatives of the U.S. Food and Drug Administration (FDA) and other federal and regulatory agencies (as required), personnel from Verily Life Sciences LLC and its affiliates (which could include Alphabet companies such as Google) and subcontractors, and the IRB may need to review your research and medical records for the purposes of this study and may learn your identity.

The payment vendor will receive information about you in order to compensate you for your participation in the study. This may include your name, email address, mailing address, and your date of birth. This information will be kept secure and confidential using data encryption methods standard to banking institutions.

Confidential information you are receiving

Signing this consent form gives Verily your permission to take your feedback and use it internally to help Verily decide how to improve its products, including use in company-wide internal communications. Participating in this study may contain confidential information so you cannot share anything you see or hear today with anyone else. In addition, if you develop any new ideas or products based on what you see or hear today, those will be the property of Verily.

De-identified Data

Verily Life Sciences LLC will use your data in the creation of a de-identified data set.

The results of this research study may be presented at scientific or medical conferences

or in publications. Your identity will not be disclosed in those presentations.

Verily Life Sciences LLC may share the data collected during this study with its affiliates (e.g., Alphabet companies, such as Google) and current and future research partners, for research, product development and other commercial purposes. Any data shared with affiliates and research partners will be de-identified and will not be linked back to your personal study participation. Verily may use the deidentified data collected during this study for other product development work within Verily.

The following shall be considered confidential information: (i) Verily's role in the study, (ii) any relationship that Verily may have with any other individuals or entities involved in the study, and (iii) the Materials (see above under Purpose of Study) provided by Verily to you. You may not talk to the media, blog, tweet, or post publicly about the Materials or your experience using the Materials, or about any other Confidential Information. You must immediately tell the study staff if you are legally required to disclose Confidential Information. You must keep all such Confidential Information secret and use it only to participate in the study.

However, Confidential Information does not include information that: (i) was known to you prior to study participation; (ii) is publicly available through no fault of yours; (iii) is rightfully received by you from a third party without a duty of confidentiality; or (iv) is independently developed by you.

You must not disclose such Confidential Information to any third party and you must use a reasonable degree of care to protect Confidential Information and to prevent any unauthorized use or disclosure of Confidential Information. You may disclose such Confidential Information when compelled to do so by law if you provide reasonable prior notice to Verily, unless a court orders that Verily not be given notice.

Unless the parties (Verily or a third party) otherwise agree in writing, your duty to protect Confidential Information survives forever.

COMPENSATION FOR INJURY

If you believe you have been injured as a direct result of the study procedures, please seek care and contact us using the information at the end of this form. There is no commitment from Verily Life Sciences LLC to provide monetary compensation or free medical care to you in the event of an injury related to a study procedure.

You will not lose any of your legal rights by signing this form.

If you think you have been injured or believe you have a research-related injury, contact the Principal Investigator and she or a member of her research team will explain to you how to obtain treatment.

VOLUNTARY PARTICIPATION AND WITHDRAWAL

Your participation in this study is entirely voluntary. You may decide not to participate or you may leave the study at any time. Your decision will not result in any penalty or loss of benefits to which you are otherwise entitled. If you decide to participate, you are free to withdraw your consent and refuse to participate at any time. You are not required to give your reason(s) for withdrawing from the study. However, the Investigator may ask for your reason(s) to exit the study while fully respecting your rights. If you decide to no longer participate in the study, no future data will be collected. Data collected up to the date you withdraw from the study will be kept and included in the study data analysis.

If you decide to withdraw from this study, please notify the Investigator at the contact address or phone number listed on page 1 of this consent form. When you withdraw from the study, no new health information identifying you will be gathered after that date. Information that has already been gathered may still be used and given to others.

You are <u>not eligible</u> to participate in this study if you are an employee, or a spouse or dependent of an employee, of Verily or an affiliate of Verily (for example, including but not limited to Google LLC, Granular Insurance Company, etc.).

Your participation in this study may be stopped at any time by the Principal Investigator or the sponsor without your consent for any reason, including:

- If you do not consent to continue in the study after being told of changes in the research that may affect you
- If the study is canceled
- If you do not fulfill the study requirements; or
- Any other reason believed to be in your best interest

SOURCE OF FUNDING FOR THE STUDY

The Sponsor, Verily Life Sciences LLC, is paying to conduct this study.

FINANCIAL DISCLOSURE

Katherine Makedonsky, OD) and the study staff are employees of the Sponsor. The Principal Investigator (PI) owns stock in Verily Life Sciences LLC. The PI's stock ownership is less than 1% of Verily Life Sciences LLC's total equity. Study staff may also own stock in Verily Life Sciences LLC. Please ask the Investigator if you have any questions relating to this potential conflict of interest.

QUESTIONS

Contact [redacted] [redacted] (24 hours) for any of the following reasons:

- if you have any questions about your participation in this study,
- if you feel you have had a research-related injury, or

- if you have questions, concerns or complaints about the research.

If you have questions about your rights as a research participant or if you have questions, concerns or complaints about the research, you may contact:

Name: WCG IRB
Address: 1019 39th Avenue SE Suite 120
Puyallup, Washington 98374-2115
Phone: 1-855-818-2289
Email: clientservices@wcgirb.com

WCG IRB is a group of people who perform independent review of research.

WCG IRB will not be able to answer some study-specific questions, such as questions about appointment times. However, you may contact WCB IRB if the research staff cannot be reached or if you wish to talk to someone other than the research staff.

Do not sign this consent form unless you have had a chance to ask questions and have gotten satisfactory answers.

If you agree to be in this study, you will receive a signed and dated copy of this consent form for your records.

CONSENT

My signature below means that:

1. I have read this consent form or it has been read to me and have had ample time to consider participation in the study.
2. I understand that I am being asked to participate in a research study, and all my questions about the study and my part in the study have been answered.
3. I freely consent to be in this research study, and voluntarily agree to comply with the study requirements.
4. I understand that I can withdraw consent at any time prior to and during the study, without any legal consequences, and without any penalty to benefits to which I am entitled.
5. I understand that my relevant personal data may be used for the purpose of this research study.
6. I agree that Verily representatives, regulatory authorities, and Institutional Review Board representatives will be granted direct access to my original medical records.
7. I will be given a signed and dated copy of this document for my records.
8. By signing this consent form, I have not given up any of my legal rights.
9. I certify that I am <u>not</u> an employee, or a spouse or dependent of an employee, of Verily or an affiliate of Verily (for example, including but not limited to Google LLC, Granular Insurance Company, etc.).

____
Participant Name (printed)

____________________________________ __________________
Signature of Participant Date

____
Printed Name of Person Conducting the
Informed Consent Discussion

____________________________________ __________________
Signature of Person Conducting the Date
Informed Consent Discussion

OPTIONAL CONSENTS

I agree to have photographs taken.

YES __________ (initial) NO ________(initial)

I agree to have videos taken.

YES __________ (initial) NO ________(initial)

ADDITIONAL / NEW STUDIES
From time to time, new studies may arise. You may be invited to take part in these studies. These studies are completely optional, and you will be given information about new studies to help you make a decision about participating. If you decide to join one of these studies, you will be asked to sign a new consent form.

I agree to be contacted about future Verily research opportunities. I understand that I can opt-out at any time by contacting the study PI or emailing vrc-feasibility-study@google.com.

YES __________ (initial) NO ________(initial)

AUTHORIZATION TO USE AND DISCLOSE INFORMATION FOR RESEARCH PURPOSES

Personal health information (PHI) is any health information that can reasonably be used to identify you, including your name, birth date, medical record number, or other personal identifying information. The Investigator and study staff may need to look at and disclose your medical and study records that contain PHI. This can include your medical health history, research records of your participation, and records about your study visits. The information necessary for evaluation of the study will be documented on special forms.

Verily Life Sciences LLC will analyze and use the information they receive for product development purposes. If necessary for these purposes, they may communicate information to affiliates of Verily Life Sciences LLC (which could include Alphabet companies such as Google), people and companies with whom Verily Life Sciences LLC works, institutional review boards (IRBs), ethics committees (ECs), the U.S. Food and Drug Administration (FDA), the Department of Health and Human Services (DHHS) agencies, and other regulatory agencies. Once your information has been disclosed to the organizations listed, it may be subject to re-disclosure and no longer subject to protection by state and federal privacy laws.

Representatives from government agencies, including the FDA, IRB, and EC, and Verily Life Sciences LLC or its agents, may also be granted direct access to your original medical records for the purpose of checking data collected for the study. Access will be granted without violating your confidentiality, to the extent permitted by applicable laws and regulations. By signing this document, you authorize this access.

You may withdraw or take away your permission to use and disclose your health information at any time. You do this by notifying the Investigator. If you withdraw your permission, your participation in the study will end and the study personnel will stop collecting your information. By signing this document, you give permission to retain, access, and use any information that has already been collected.

The results of this study may be published or discussed in conferences; however, no information will be included that would readily reveal your identity.

You have the right not to sign this authorization, but if you do not sign it, you cannot participate in the research study. Refusal to sign this authorization will not result in a loss of any benefit to which you are entitled. This permission will be good until **January 1, 2026**. You have a right to receive a copy of this authorization.

SIGNATURE OF RESEARCH PARTICIPANT

By signing this authorization, I willingly agree to permit my information to be used and disclosed as described above.

____
Participant Name (printed)

____ __________________
Signature of Participant Date
